# Institutional Questions

**Published:** 1900-05-19

**Authors:** 


### INSTITUTIONAL QUESTIONS.

**Hospital Slop-Sink.**

A Correspondent asks: What is the best form of slop-sink for hospital use? If you can give me information and advice on this point I shall be much obliged.

*** Many slop-sinks have been devised with the object of providing for the automatic cleansing of bed-pans, &c. One of the best is undoubtedly the one now very generally in use, made by Messrs. Dent and Hellyer. You should get and read a little pamphlet on the "Sanitation of Hospitals," by Mr. Keith D. Young, F.R.I.B.A., which gives much useful information on all such matters. He thus describes this sink and its method: "The bed-pan is placed face downwards, with its nozzle inclining towards the outlet of the sink; a jet of water is then projected upwards from the rose underneath the sink and flushes out the whole pan, the pan itself being held in position by the india rubber strips at the sides of the sink. To flush out the sink there is a flushing rim, the water to which is turned on by a separate handle. There is a separate jet for flushing out urinals, and a metal cradle was devised for holding these vessels; but the latter is an encumbrance, and is not at all necessary. The room in which this sink is fixed should be of sufficient size to hold not only the slop sink itself, but an ordinary porcelain sink for washing other vessels, such as spittoons, porringers, &c., and sufficient shelving to hold the ward crockery."

**The Crusade against Consumption.**

"Interested" writes: Can you tell me anything about the association which has been formed with the object of working against the spread of consumption, what it does, and where its offices, if any, are to be found? Also I want to know whether the latest returns with regard to this disease show an increase or a decrease.

*** The National Association for the Prevention of Consumption and other forms of Tuberculosis has been in existence for some little time, and communications should be addressed to the secretary, 20, Hanover Square, W. The objects with which this society was started are best expressed by the following manifesto, issued under its name:—

1. Object.—The prevention of tuberculosis.
2. Membership.—The association consists of ordinary and of life members. The subscription of ordinary members is 5s. annually. Life members give a donation of five guineas.
3. Methods.—
   I. The education of public opinion and the stimulation of individual initiative by means of
      (a) A central bureau for the collection and distribution of information as to modes of diffusion of tuberculosis and measures of prevention.
      (b) The circulation of pamphlets and leaflets setting forth in plain language the results of scientific investigation of the above points.
      (c) Public lectures by men approved by the council; addresses at congresses and other public gatherings.
      (d) Co-operation with other societies having for their object the promotion of public health.
      (e) The co-operation of the public press.
      (f) The holding of periodical congresses, and the issue of an annual report.
      (g) The promotion of the establishment on a self-supporting basis of open-air sanatoria for tuberculosis patients.
   II. The influencing of Parliament, County Councils, boards of guardians, and other public authorities on matters relating to the prevention of tuberculosis.

The figures you want are to be found in the annual report of the Registrar-General of Births, Deaths, and Marriages, to be obtained from Messrs. Eyre and Spottiswoode, East Harding Street, E.C. In the report for 1897 it was shown that "among persons at all ages and of both sexes, the mortality from phthisis has decreased very considerably; but that among females the decrease has been much greater than among males. Among the former the aggregate mortality from phthisis in 1891-97 was scarcely more than half of what it had been in 1861-70. Among the latter the decrease amounted to rather more than one-third."

**Hospitals and the Limited Liability Act.**

A Correspondent writes: A proposal has been made that a hospital with which I am connected should be incorporated under the Companies' Acts of 1862 and 1867 for the purpose of facilitating business transactions, and I should be much obliged if you would kindly tell me if this plan has as yet been adopted in any other hospital, and if so, how it has worked? It has been done, I know, in the case of educational institutions, such as some University colleges, and I believe also in the case of some charities. I have not, however, heard of any hospital which has been incorporated.

*** You will find a chapter on incorporation in the "Legal Handbook for the Use of Hospital Authorities," by L. S. Bristowe, which you can obtain, we believe, from the Scientific Press, 28, Southampton Street, Strand, London, or from any bookseller, price 3s. 6d. The Home Hospitals Association for Paying Patients, Fitzroy House, Fitzroy Square, London, N.W. (hon. secretary, T. Almond Hind), is incorporated under the Limited Liability Acts by licence from the Board of Trade to dispense with the word "limited." The usual procedure for a hospital is to obtain a Royal Charter. The Royal Free Hospital, Gray's Inn Road, London, W.C., have thus incorporated so recently as 1892, and the secretary, Mr. C. W. Thies, would no doubt give you any particulars. No special form of procedure is prescribed in regard to incorporation by Royal Charter, and all information can be obtained from the Clerk to the Privy Council, at the Privy Council Office, Whitehall, London, S.W.

